# Tailoring Polarization Conversion in Achiral All-Dielectric Metasurfaces by Using Quasi-Bound States in the Continuum

**DOI:** 10.3390/nano12132252

**Published:** 2022-06-30

**Authors:** Jose Luis Pura, Ruhinda Kabonire, Diego R. Abujetas, José A. Sánchez-Gil

**Affiliations:** 1 Instituto de Estructura de la Materia (IEM-CSIC), Consejo Superior de Investigaciones Científicas, Serrano 121, 28006 Madrid, Spain; kpruhinda@gmail.com (R.K.); diego.romeroabujetas@unifr.ch (D.R.A.); 2 Dipartimento di Ingegneria dell’Informazione (DEI), Università Degli Studi di Padova, Via Gradenigo 6/b, 35131 Padova, Italy; 3 Physics Department, Fribourg University, Chemin de Musée 3, 1700 Fribourg, Switzerland

**Keywords:** all-dielectric metasurfaces, bound states in the continuum, resonances, coupled-dipole theories, polarization conversion

## Abstract

Quasi-bound states in the continuum (quasi-BICs) supported in all-dielectric metasurfaces (MTS) are known for their confinement in real space and the notably high values of the quality factor Q. Recently, the properties of quasi-BICs have been employed to achieve polarization conversion with all-dielectric MTS. However, one of the main disadvantages of the current approaches is the dependence on the chirality of either the meta-atoms or their disposition. We present the possibility of achieving polarization conversion by using all-dielectric MTS with square and rectangular lattices of nano-disks. The precise tuning of the lattice and disks parameters allows to transform linearly polarized light into circularly polarized light with near unity polarization rates while maintaining the high Q values of quasi-BICs. Moreover, by using double accidental BICs it is possible to obtain right and left circularly polarized light on demand just by varying the angle of incidence.

## 1. Introduction

All-dielectric metasurfaces (MTS) are seen as the future of traditional optical devices. The technological developments over recent years have expanded their potential to a diversity of applications [1]. The possibility of joining their unique photonic capabilities together with their ultra-compact design leads the way to the creation of innovative integrated optical systems [2]. Additionally, the dielectric nature of all-dielectric MTS results in a nearly lossless optical response. This makes them particularly attractive as an alternative to lossy plasmonic nanostructures, while maintaining the ability to control light at the nanoscale [3].

Controlling light polarization with all-dielectric MTS can be achieved by a wide variety of methods [4,5,6,7,8]. The later advances have boosted the potential applications of metasurfaces by allowing features such as broadband polarization [9], which is fundamental for the fabrication of polarizers and waveplates, or new approaches for the production of polarization detectors [10,11]. More detailed control of the polarization states led to the design of advanced devices such as polarization beam splitters [12,13], which can be employed in polarization imaging and telecommunications, vector vortex beams [14] or multifunctional devices based on the combination of several physical properties involving light polarization [15,16], among others [17].

A truly interesting phenomenon that might arise in all-dielectric MTS is the occurrence of bound states in the continuum (BICs). Perfect BICs present a theoretically infinite quality factor Q, which results in their complete isolation from the surrounding radiation states [18,19,20,21,22,23]. There are two main types of BICs supported by all-dielectric MTS: symmetry-protected and accidental BICs (aBICs) [24]. Symmetry protected BICs can only occur at normal incidence (i.e., Γ point of the reciprocal space), while aBICs appear for non-zero values of the wavevector in-plane component [25,26,27]. Aside from their interesting properties, the perfect isolation of BICs from the radiation continuum makes it virtually impossible to achieve any physical interaction with such states, hindering their straightforward use in practice. The typical approach to bypass this drawback aims to break the symmetry conditions that make the BIC possible, either in a controlled manner [28,29,30,31,32] or simply by taking advantage of the natural imperfections of the real fabrication process. This symmetry rupture process results in the generation of the so-called quasi-BICs. Quasi-BICs present high but finite values of the Q factor while maintaining most of the properties of the perfect BIC. The key point lies in the finite value of Q which permits the interaction of the quasi-BIC with the radiative continuum.

The optical response of BICs has recently been harnessed to achieve polarization conversion [33,34,35,36]. However, one of the main drawbacks of the current approaches is the dependence on the intrinsic chirality of the meta-atoms, or their disposition in chiral arrangements. These conditions translate into considerable fabrication challenges, especially for radiation in the visible spectrum. The present work shows the possibility of achieving polarization conversion by using quasi-BICs of all-dielectric MTS with remarkably simple geometries: square and rectangular lattices of nano-disks. On the other hand, the use of quasi-BICs comes at the price of resigning from using the normal incidence geometry, which could be undesirable for certain applications. Finally, the precise tuning of the lattice parameters allows to transform linearly polarized light into circularly polarized light with near unity polarization rates.

## 2. Materials and Methods

The calculation of the optical response of the considered metasurfaces is based on the coupled electric and magnetic dipole (CEMD) formalism previously developed [37,38]. This formalism provides both the reflection and transmission coefficients of the metasurface under study for plane wave incidence [38], as well as the near fields and local density of states if a point dipole source is considered [39]. Quite importantly, the resulting quasi-analytical calculations, orders of magnitude faster than numerical simulations, exhibit not only qualitative but also quantitative accuracy, inasmuch as only dipolar modes come into play; indeed, our CEMD model has been successfully exploited to reproduce/predict BIC-related phenomenology in a variety of metasurfaces throughout the electromagnetic spectrum [22,32]. Other coupled-dipole theories have been successfully exploited to deal with a variety of particle arrays [40,41,42,43,44,45].

The electromagnetic field components are given as 6 × 1 column vectors of the form:

(1) $$E=\left(\begin{array}{c} E_{x}\\ E_{y}\\ E_{z}\\ ZH_{x}\\ ZH_{y}\\ ZH_{z} \end{array}\right),$$

where $E_{i}$ and $H_{i}$ are the Cartesian components of the electric and magnetic fields, respectively, and *Z* is the intrinsic impedance of the surrounding medium, given as $Z=\sqrt{\mu /\epsilon }$, ϵ and μ being the medium electric permittivity and magnetic permeability, respectively. In the absence of a substrate the impedance will be that of vacuum for incident, reflected and transmitted waves.

Once the fields are known the calculation of the reflection and transmission amplitudes is straightforward for both transversal electric (TE) and transversal magnetic (TM) modes,

(2) $$\begin{array}{cccc} r_{TE} & =-\frac{E_{y,r}}{E_{y,i}}, & t_{TE} & =\frac{E_{y,t}}{E_{y,i}},\\ r_{TM} & =-\frac{H_{y,r}}{H_{y,i}}, & t_{TM} & =\frac{H_{y,t}}{H_{y,i}}, \end{array}$$

where the indexes r/t/i denote the reflected, transmitted and incident fields, respectively. The assignment of the minus signs could change depending on the selected system of reference. For calculation purposes the incident field amplitude can be set to unity (giving the proper attention to relative field orientation) which further simplifies Equation (2).

### 2.1. Calculation of the Stokes Parameters

The Stokes parameters can be calculated for both the transmitted and reflected fields. In this work, reflection will be investigated since a sharp peak in reflectance is observed near the BIC incident angle and wavelength parameters. However, in transmission a reduction of the transmittance appears, resulting in a reduced amount of available light. Every calculation performed for reflection can be directly translated to transmission without loss of generality.

**Figure 1:**
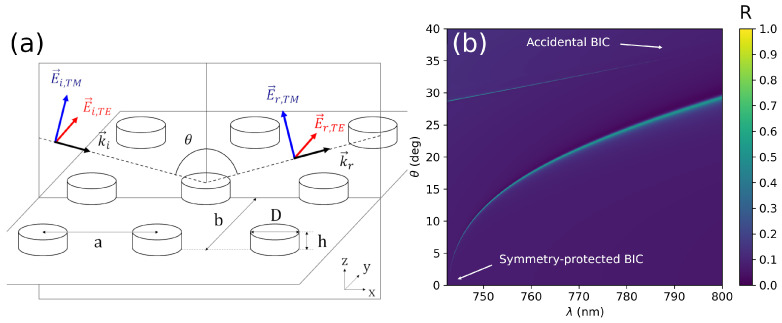
(**a**) Scheme of the geometrical parameter of the considered MTS and the possible field polarizations. (**b**) Reflectance map of a square array of poly-Si disks (h=80 nm, D=220 nm), the lattice parameter being a=340 nm. There are two high reflectivity regions associated to two different BICs. The first BIC (symmetry-protected) is located at normal incidence θ=0° and it is associated to a TE mode. The second one is an aBIC located close to 37° and 800 nm and presents TM polarization.

Stokes parameters can be calculated in the light system of reference by using the following expressions:

(3) $$\begin{array}{cc} I & =|E_{TE}{|}^{2}+|E_{TM}{|}^{2},\\ Q & =|E_{TE}{|}^{2}-|E_{TM}{|}^{2},\\ U & =2Re\left(E_{TE}E_{TM}^{*}\right),\\ V & =2Im\left(E_{TE}E_{TM}^{*}\right), \end{array}$$

where $E_{TE}$ and $E_{TM}$ stand for the electric field components along the TE and TM polarization directions, respectively, see the scheme in Figure 1a. The TE is equivalent to the *x* axis of the MTS reference system in this configuration, and the TM direction is contained in the scattering plane and it is orthogonal to the *x* axis and the wavevector ${\vec{k}}_{i}$.

The Stokes parameters contain complete information of the polarization state. The *I* parameter provides information about the total degree of polarization. In this context it will be equivalent to either the reflectance or the transmittance, depending on the studied case, since the amplitude of the incident field is set to unity. The *Q* parameter informs about the proportion of Cartesian polarized light in the selected base; in this case it will correspond to the ratio of pure TE (Q>0) or TM (Q<0) polarization. The *U* parameter provides the amount of linearly polarized light in the two orthogonal diagonal directions, i.e., 45° (U>0) and 135° (U<0) with respect to TE polarization. It is also useful to define a parameter to hold the total proportion of linear light regardless of its particular orientation inside the polarization plane: $L=\sqrt{Q^{2}+U^{2}}$. Finally, the *V* parameter contains the information of the circularly polarized light and its helicity, right circular polarization (RCP) light (V>0) and left circular polarization (LCP) light (V<0). Note that the condition that must be satisfied by the Stokes parameters for coherent monochromatic light is the following:

(4) $$I^{2}=Q^{2}+U^{2}+V^{2}=L^{2}+V^{2}.$$

It is common to normalize Q,U,L and *V* by *I* to obtain the contributions of each polarization with respect to the radiation that is being studied (reflected or transmitted). By doing this, $L_{n}^{2}=L^{2}/I^{2}$ and $V_{n}^{2}=V^{2}/I^{2}$ reflect the proportion of each polarization (linear and circular) and are forced to sum up to unity.

In order to analyze the polarization changes the incident field polarization is set to linearly polarized light at 45° with respect to both TE and TM polarizations, i.e., $E_{i}\;=\;\left(1/\sqrt{2},-1/\sqrt{2}\right)$ in the TE/TM framework. Then, the reflected field takes the form $E_{r}=\left(r_{TE}/\sqrt{2},-r_{TM}/\sqrt{2}\right)$, and can be substituted in Equation (3) to obtain the Stokes parameters of the reflected light.

### 2.2. Nanodisk Array Implementation

The cases under study comprise two different arrays of high refractive index poly-crystalline Si (poly-Si) nanodisks, similar to those used in the experimental work in Ref. [46]; as measured therein, it should be emphasized that, in the spectral region of interest, including part of the visible domain, poly-Si has a large refraction index $n_{pSi}≃4$, with nearly negligible absorptive losses ($k_{pSi}\approx 0$). In order to perform the calculations, the CEMD model must be fed with the necessary physical properties: the array lattice constants *a* and *b*, the background (relative) electrical permittivity $\varepsilon_{b}$ and magnetic permeability $\mu_{b}$, and the polarizability tensor of the constituent atoms $\overset{↔}{\alpha }$. A scheme of the MTS with the relevant geometrical quantities is provided in Figure 1a.

In the first studied case, the metasurface is formed by a square array of poly-Si disks, a=b=340 nm; they are embedded in an index matching material with $\varepsilon_{b}=2.1$, $\mu_{b}=1$. The polarizability tensor $\overset{↔}{\alpha }$ is numerically computed for poly-Si disks 80 nm in height and 220 nm in diameter by using SCUFF, a free software implementation based on the method of moments [47].

In the second case, the disks array is rectangular with a=455 nm, b=845 nm; the disks are embedded in vacuum $\varepsilon_{b}=\mu_{b}=1$. In this case, the polarizability tensor $\overset{↔}{\alpha }$ is computed for poly-Si disks 200 nm in height and 210 nm in diameter.

In both cases, our CEMD has revealed qualitative and quantitatively accurate to reproduce either experimental extinction [46] or numerical simulations [27], since no higher order multipoles come into play in the spectral regions of interest for those nanodisks; if higher frequency regions were to be studied, quadrupolar or higher terms could be added as in, e.g., Refs. [42,48,49].

## 3. Results and Discussion

### 3.1. Single BICs

The first section of the results includes the simplest case of BIC-induced polarization conversion. The system comprises a square array of poly-Si disks, previously defined. Figure 1b presents the total reflection factor $R=(|r_{TE}{|}^{2}+|r_{TM}{|}^{2})/2$ for this system showing two well separated modes. The first (symmetry-protected) BIC is located near 740 nm at normal incidence (θ=0°), presents TE polarization and transforms into a quasi-BIC when increasing θ. The second one is an aBIC with TM polarization near 800 nm and θ≃37°.

**Figure 2:**
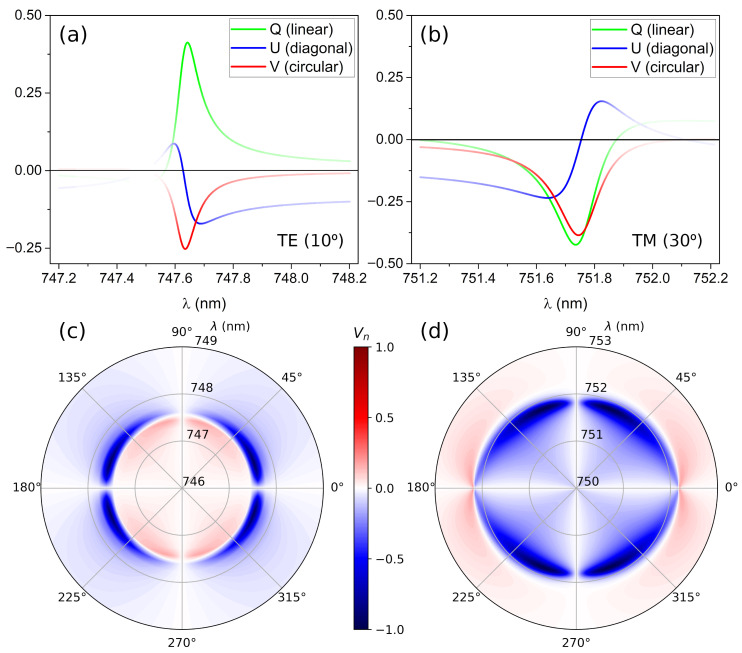
Stokes parameters of the reflected light as a function of the incident wavelength for (**a**) the TE quasi-BIC at θ = 10°, and (**b**) the TM quasi-BIC at θ = 30° for incident light polarized at 45°. The line intensity is proportional to the *I* parameter (i.e., reflected light intensity). The behavior is notably similar, presenting opposite signs for the *Q* parameter, which is positive for TE and negative for TM. However, the *V* parameter is always negative and maximum in absolute value at the BIC location, which implies the attaining of LCP light regardless of the TE/TM nature of the considered BIC. Figures (**c**,**d**) represent a polar plot of the $V_{n}$ parameter as a function of the incident polarization angle and wavelength (radial axis) for the TE and TM quasi-BICs.

The Stokes parameters of the reflected light can be calculated in the proximity of the two quasi-BICs. The results are summarized in Figure 2. The Stokes parameters are plotted in Figure 2a,b for a fixed angle as a function of the incident wavelength for incident light polarized at 45°. Both TE and TM quasi-BICs present the expected asymmetric Fano shape [46]. *I* and *Q* parameters help to locate the quasi-BIC position, the *Q* parameter being positive for TE and negative for TM cases, as expected. The high absolute value of the *Q* parameter implies that the quasi-BIC reflects the light with matching polarization. Analyzing the two remaining Stokes parameters, *U* and *V*, it can be seen that *U* always crosses zero at the quasi-BIC location; however, the *V* parameter is always negative at that point, which implies the attaining of LCP light by reflection. Furthermore, in this case the polarization is always LCP regardless of the TE/TM nature of the BIC under study. Figure 2c,d represent the $V_{n}$ parameter as a function of the polarization angle and wavelength (radial axis) for the TE and TM quasi-BICs. There is a highly localized region producing LCP light as previously observed. It is interesting to note that the maximum of the $V_{n}$ parameter does not happen for incident light at 45° but it is shifted towards 0° in the case of the TE quasi-BIC and 90° for the TM quasi-BIC. This is a result of the higher reflectivity of the MTS for the light matching the quasi-BIC polarization.

### 3.2. Double Accidental BIC

**Figure 3:**
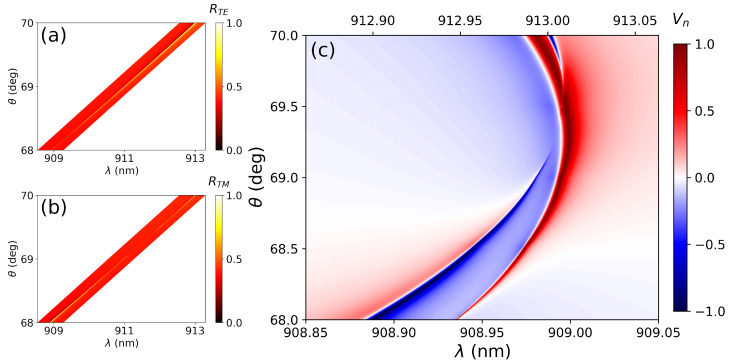
(**a**) Reflectance map of the TE aBIC. (**b**) Idem for the TM aBIC. (**c**) Representation of the normalized *V* stokes parameter $V_{n}$ as a function of the wavelength and incident angle for the double aBIC. The plot is an expansion of the parallelogram region on Figures (**a**,**b**) to allow a better visualization. As a result, the *x*-axis changes as a linear function of the incident angle.

The study is based on a previously proposed system that exhibits the exact coincidence of two aBICs [27]. In this system the occurrence of BICs takes place at certain incident angles and wavelengths for which dipole interference prevents radiation leakage to the continuum, thereby being called accidental BICs. For the present work, the lattice constants have been slightly modified to prevent the co-occurrence of the two aBICs and allow their overlapping on the quasi-BIC region. By doing this, the Stokes parameters can be better resolved with less computational effort, and at the same time the results will be similar to that of a real system. The results for this arrangement can be found in Figure 3. Figure 3a,b present reflectance maps of the TE and TM aBICs, respectively. It can be seen that the positions of the two exact aBICs do not coincide, as planned. Figure 3c presents the normalized *V* stokes parameter $V_{n}$ as a function of the wavelength and incident angle for the double aBIC. The plot is an expansion of the parallelogram regions depicted on Figure 3a,b to allow for a better visualization of the results. As a result, the *x*-axis of the plot changes as a linear function of the incident angle. There are two clearly distinguished regions with opposite values of *V*. In the region close to θ=68° and λ=908.90 nm, the value of $V_{n}$ closely approaches −1. This means that, at those conditions, the metasurface will be largely reflecting LCP light. On the other hand, for the region closer to θ=70° and λ=913 nm $V_{n}$ approaches +1 and the reflected radiation will be mainly composed by RCP light.

**Figure 4:**
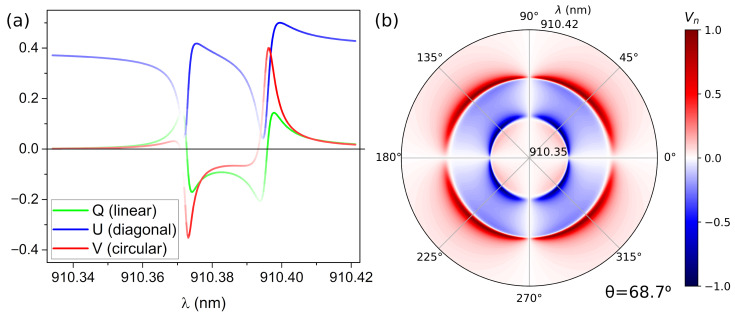
(**a**) Stokes parameters of the reflected light as a function of the incident wavelength for the double aBIC (θ=68.7°). The line intensity is proportional to the I parameter (i.e., reflectance). (**b**) Polar plot of the $V_{n}$ parameter in reflection as a function of the incident polarization angle and wavelength (radial axis) for the double aBIC (θ=68.7°).

Figure 4 presents a detail of the polarization features at θ=68.7°. Figure 4a shows the Q, U and V Stokes parameters, the line intensity being proportional to the I parameter (i.e., the reflectance). Figure 4b shows a polar plot of the $V_{n}$ parameter in reflection as a function of the incident polarization angle and wavelength (radial axis). At lower wavelength values we can observe the generation of LCP light by the TM aBIC (blue lobes), whereas for higher wavelength values RCP light is reflected by the TE aBIC (red lobes). It is interesting that the shift of the maximum from 45° is not as pronounced as in the square lattice.

This second metasurface is significantly different from the previous one for two reasons. Firstly, it presents the possibility of obtaining the two different orientations for the circular light in reflection, rather than the first case that only allows obtaining LCP light. Secondly, the proximity of both states in wavelength and incident angle would permit to switch from RCP light to LCP light just by varying the angle of incidence in ≈2°. Another practical approach, as presented in Figure 4, could be the generation of both RCP and LCP light at two close wavelengths for a fixed incident angle. The election for the optimum implementation will depend on the final application.

As a summary, all the polarization and spectral parameters of the considered cases are collected in Table 1.

## 4. Conclusions

The polarization conversion by using BICs on all-dielectric MTS without either chiral nanoparticles or chiral arrangements has been shown to be possible. The tailoring of BICs naturally occurring in all-dielectric MTS allows for the transformation of linearly polarized light into circularly polarized light by reflection. This could be obtained by using relatively simple arrangements of nano-disks. Furthermore, the occurrence of double aBICs results in the possibility of obtaining RCP and LCP light on demand.

All of these possibilities come together with excellent technical capabilities. On one hand, the degree of circular polarization provided by the $V_{n}$ Stokes parameter can reach values highly close to unity for the double aBIC system. On the other hand, the spectral linewidth of the studied cases ranges from around 0.2–2 nm, for the single TE and TM modes, to 6 × 10${}^{-3}$ nm, for the double aBIC case. This is naturally linked to the substantially large quality factors of quasi-BICs. Moreover, the linewidth could be finely tuned by changing the quasi-BIC profile simply by modifying the angle of incidence.

This polarization and spectral remarkable properties could pave the way for the fabrication of ultra-compact nanophotonic devices joining polarization conversion capabilities with excellent and tunable spectral resolution. Furthermore, the same concept could be extrapolated to any other (lower frequency) region of the electromagnetic spectrum.

## Figures and Tables

**Table 1:** Polarization and spectral parameters of all the studied cases. The values of the Stokes parameters are calculated for linearly polarized light at 45°.

	R	$L_{n}^{2}$	$V_{n}^{2}$	λ/nm	Δλ/nm	Q-Factor
TE (10°)	0.145	0.567	0.433	747.588	0.139	5375
TM (30°)	0.397	0.490	0.510	751.795	0.314	2391
aBIC RCP	0.410	5 × 10${}^{-6}$	0.999995	911.119	0.006	158,016
aBIC LCP	0.375	10 × 10${}^{-6}$	0.999990	909.339	0.008	113,667

## Data Availability

The data that support the findings of this study are available from the corresponding author upon reasonable request.

## Abbreviations

The following abbreviations are used in this manuscript:

MTS	Metasurfaces
BIC	Bound state in the continuum
aBIC	Accidental BIC
CEMD	Coupled electric & magnetic dipole
TE	Transversal electric
TM	Transversal magnetic
RCP	Right-handed polarization
LCP	Left-handed polarization
